# Deep Learning for Breast Cancer Diagnosis from Mammograms—A Comparative Study

**DOI:** 10.3390/jimaging5030037

**Published:** 2019-03-13

**Authors:** Lazaros Tsochatzidis, Lena Costaridou, Ioannis Pratikakis

**Affiliations:** 1 Visual Computing Group, Department of Electrical and Computer Engineering, Democritus University of Thrace, 67100 Xanthi, Greece; 2 Department of Medical Physics, School of Medicine, University of Patras, 26504 Patras, Greece

**Keywords:** mammography, breast cancer, deep learning, convolutional neural networks, CAD

## Abstract

Deep convolutional neural networks (CNNs) are investigated in the context of computer-aided diagnosis (CADx) of breast cancer. State-of-the-art CNNs are trained and evaluated on two mammographic datasets, consisting of ROIs depicting benign or malignant mass lesions. The performance evaluation of each examined network is addressed in two training scenarios: the first involves initializing the network with pre-trained weights, while for the second the networks are initialized in a random fashion. Extensive experimental results show the superior performance achieved in the case of fine-tuning a pretrained network compared to training from scratch.

## 1. Introduction

Recent studies have shown that breast cancer is the most common type of cancer among women [1], accounting for about one third of newly diagnosed cancers in the US [2]. The mortality rate of breast cancer is also high, accounting for 17% of deaths relating to cancer in general [3]. Accurate detection and assessment of breast cancer in its early stages is crucial when it comes to reducing the mortality rate. Mammography is until today the most useful tool for general population screening. However, the accurate detection and diagnosis of a breast lesion solely based on mammography findings is difficult and highly depends on the expertise of the radiologist, which leads to a high number of false positives and additional examinations [4].

Computer-aided detection and diagnosis (CAD) systems are already being used to offer crucial assistance in the decision-making process of radiologists. Such systems may significantly reduce the amount of effort needed for the assessment of a lesion in clinical practice, while minimizing the number of false positives that lead to unnecessary and discomforting biopsies. CAD systems regarding mammography may address two different tasks: detection of suspicious lesions in a mammogram (CADe) and diagnosis of detected lesions (CADx), i.e., classification as benign or malignant.

Deep learning is considered a significant breakthrough technology of recent years as it has exhibited performance beyond the state-of-the-art in various machine learning tasks including object detection and classification. Contrary to conventional machine learning methods, which require a hand-crafted feature extraction stage, which is challenging as it relies on domain knowledge, deep learning methods adaptively learn the appropriate feature extraction process from the input data with respect to the target output. This eliminates the tedious process of engineering and investigating the discrimination ability of the features while facilitating the reproducibility of the methodologies.

Since the emergence of deep learning, various works have been published exploiting deep architectures [5]. The most common type of deep learning architecture is the convolutional neural network (CNN). Arevalo et al. [6] tested various CNNs and compared them with two hand-crafted descriptors for the task of mass diagnosis. Their experimentation was conducted on the BCDR-FM dataset. They reported performance improvement with the combination of both learned and hand-crafted representations. However, the authors did not test the performance of pretrained networks and used simpler CNN architectures. Carneiro et al. [7] used a pre-trained CNN that was fine-tuned using unregistered mammograms and segmented microcalcification and masses. They estimated the risk of developing breast cancer according to BIRADS. They concluded that the pre-trained models are superior to the randomly initialised ones. Huynh et al. [8] used the AlexNet [9] pre-trained without further fine-tuning it for the problem of mass diagnosis. They analyzed the performance of classification using features from various intermediate layers of the network using SVM for the classification. They compared their results to two approaches: a classifier operating on hand-crafted features and an ensemble of both, using soft voting. Jiao et al. [10] proposed a scheme in which a pre-trained CNN was fine-tuned on a subset of DDSM database. Then, features of masses were extracted from different layers of this model. In this way ‘high-level’ and ‘middle-level’ features were obtained that correspond to different scales. Two linear SVM classifiers are trained for the decision procedure, one for each feature group, and their predictions are fused. Levy and Jain [11] classified mammographic masses using AlexNet and GoogleNet. They compared transfer learning to from-scratch training finding that the former achieves superior results. It is worth noting that they investigated the effect of data context, concluding that cropping larger bounding boxes of fixed size around the lesion is more effective compared to cropping with proportional padding. Ting et al. [12] created and trained from scratch their network for breast mass classification. Their network comprises 28 convolutional and fully-connected layers and it is fed by proposal ROIs detected by an one-shot detector. They conducted their experiments in MIAS database. Rampun et al. [13] used an ensemble of a slightly modified version of AlexNet pre-trained and fine-tuned on CBIS-DDSM. During inference, they picked the three best performing models and fused their predictions.

The majority of state-of-the-art works proposes the utilization of pre-trained networks against training from scratch. However, state-of-the-art networks are designed and tested on much more diverse datasets, different in nature and several orders of magnitude larger than the available mammographic datasets. Consequently, the capacity and complexity of such networks may by far exceed the needs of smaller datasets, leading to major adverse effects when training from scratch. As a result, several works have appeared whose authors propose from-scratch training.

Considering the above, in this paper we investigate the performance of multiple networks. We compare the performance of each network in two scenarios: the first scenario involves initiating the training with pre-trained weights while for the second the networks are initialized with random weights.

The remainder of the paper is organized as follows: Section 2 details the deep learning architectures used in the current study for the classification of mammograms as benign or malignant. Section 3 presents the experimental setup along with the corresponding results. Finally, in Section 4 conclusions are drawn.

## 2. Methodology

### 2.1. Convolutional Neural Networks

#### 2.1.1. AlexNet

**Figure 1:**
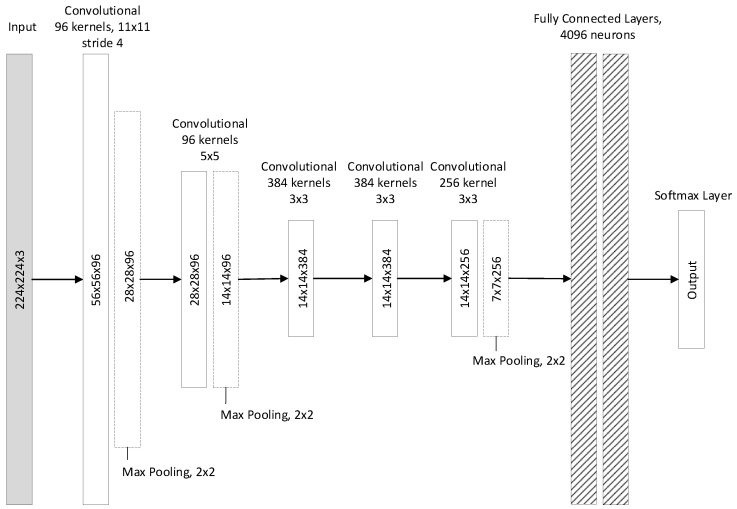
AlexNet structure.

AlexNet [9] was the first convolutional neural network (CNN) that exhibited performance beyond the state-of-the-art in the task of object detection and classification. As shown in Figure 1, the network contains eight layers; the first five are convolutional an the remaining three are fully-connected. The first layer of the network filters the input image (sized 224×224) with 96 kernels of size 11×11 with a stride of 4 pixels. The depth of these kernels equals the number of channels of the input image. The second layer takes as input the output of the first layer, after local response normalization and max-pooling have been applied, filtering it with 256 kernels of size 5×5×96. The third, fourth and fifth layers are connected to one another without any intervening pooling or normalization layers. The third layer has 384 kernels of size 3×3×256. The fourth layer has 384 kernels of size 3×3×384 and the fifth layer has 256 kernels of size 3×3×384. On top of the convolutional layers, two fully-connected layers are connected that have 4096 neurons each. The number of neurons in the third fully-connected layer equals the number of classes.

Along with the particular architecture of the network, the authors of [9] also introduced some novel features that greatly contribute to the network’s ability to learn and generalize. The most important feature is that they replaced the standard neuron activation functions (logistic function and hyperbolic tangent) with the rectified linear function f(x)=max(0,x). Neurons that use this activation function are referred to as Rectified Linear Units (ReLUs). The advantage of this activation function is that it imposes a non-saturating non-linearity in contrast to sigmoid functions that saturate for large values. This allows for significantly better flow of the gradients along with improved calculations efficiency. ReLUs are established as the standard choice of activation function for CNNs. The authors also introduced a depth-wise normalization scheme for each location of the feature maps produced by a convolutional layer, called Local Response Normalization (LRN). This sort of response normalization creates a competition for big activities amongst neuron outputs computed using different kernels. While LRN was adopted and incorporated into various other network architectures, it was removed from AlexNet in a subsequent publication [14].

A particularly important aspect of training was the use of dropout [15] (with probability of 0.5) for the three fully-connected layers. This technique consists of setting to zero the output of each hidden neuron with some probability. The neurons that are picked contribute to neither the forward pass nor the back-propagation. Thus, in every training iteration, a different architecture is sampled. The dropout technique acts as a regularizer, forcing the network to learn meaningful features, but increases the training time.

#### 2.1.2. VGG

In [16] authors investigated the effect of network depth while keeping the convolution filters very small. They showed that significant improvement can be achieved by pushing the depth to 16–19 layers. The input to the convolutional layer is a fixed-size 224×224 image. The image is passed through a stack of convolutional layers with ReLU activations where filters with very small receptive fields (3×3) were used. The convolution stride is also fixed to 1. Spatial pooling is carried out by five max-pooling layers, performed after some of the convolutional layers. Similarly to AlexNet, a stack of three fully-connected layers is put on top of the convolutional part of the network. The advantage of VGG is that, by stacking multiple convolutional layers with small-sized kernels, the effective receptive field of the network is increased, while reducing the number of parameters compared to using less convolutional layers with larger kernels for the same receptive field.

**Figure 2:**
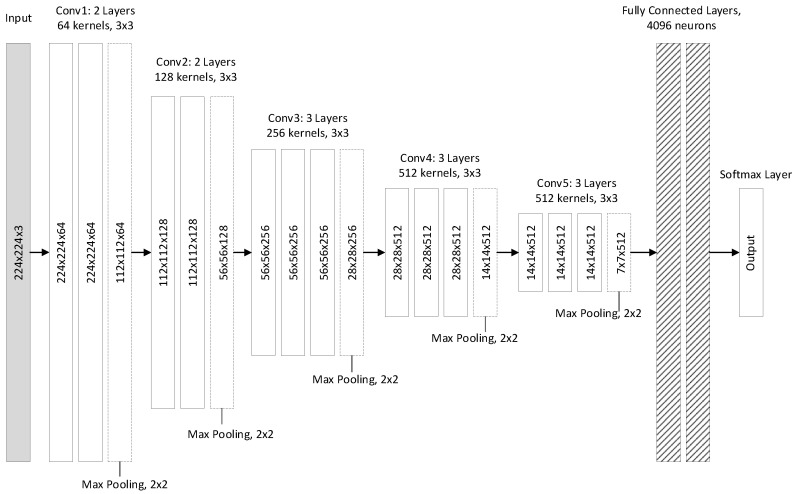
VGG-16 structure.

The authors tested multiple configurations of varying depth (9,11,16, and 19 layers). In one of the configurations 1×1 filters were utilized, which can be seen as a linear transformation of the input channels. This is also a way to increase the non-linearity of the decision function without affecting the receptive fields of the convolutional layers. One of the configurations also included a LRN layer. As reported in the paper, best results were achieved for depths between 16 and 19. The architecture of VGG-16 is depicted in Figure 2.

#### 2.1.3. GoogLeNet/Inception

**Figure 3:**
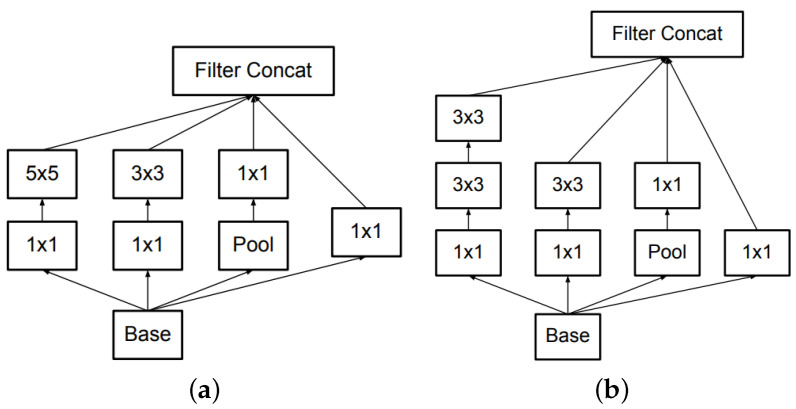
(**a**) Inception module of GoogLeNet; (**b**) Inception-v2 module.

GoogLeNet [17] is the first implementation using the Inception module. The main idea behind this module is based on authors’ findings about how a local sparse structure can be approximated by dense components. Their aim was to find the optimal local structure and repeat it, constructing a multi-layer network. The Inception module comprises four branches that get the same input (Figure 3a). The first branch filters the input with a 1×1 convolution, which acts as a linear transformation on input channels. The second and third branches perform 1×1 kerneled convolutions for dimensionality reduction followed by convolutional layers with kernels of size 3×3 and 5×5, respectively. The fourth branch performs max-pooling followed by convolution with 1×1 kernels. Finally, the outputs of each branch are concatenated and fed as input to the next block. GoogLeNet is constructed by stacking nine Inception modules. In selected locations a max-pooling layer is placed between inception modules in order to decrease the dimensionality of the feature maps. A feature of GoogLeNet worth noting is the incorporation of auxiliary classifiers. Based on the assumption that middle layers of a CNN should produce discriminative features, the authors added simple classifiers (two fully connected and a softmax layer) that operate on the features produced by an intermediate point of the network. The loss calculated by the decisions of these classifiers is used during the back-propagation stage to calculate additional gradients that contribute to the training of the respective convolutional layers. At inference time the auxiliary classifiers are discarded.

In subsequent publications [18], a revised version of the Inception module have been proposed, along with slightly modified network architectures. The authors proposed Batch Normalization (BN) and incorporated it into the Inception network. BN is a technique that makes normalization part of the model architecture, performing the normalization for each training mini-batch. The authors argue that BN allows for higher learning rates and simpler initialization techniques without experiencing adverse effects. According to BN, all the images of the current mini-batch are rescaled so that they have mean value of 0 and variance of 1. Consequently, a linear transform is applied, the parameters of which are learned through the training process. The network that was used in [18], namely Inception-v2, was a slight modification of GoogLeNet. Apart from the incorporation of BN, the most important change is that the 5×5 convolutional layers of the Inception module were replaced by two consecutive 3×3 layers (Figure 3b).

#### 2.1.4. Residual Networks

**Figure 4:**
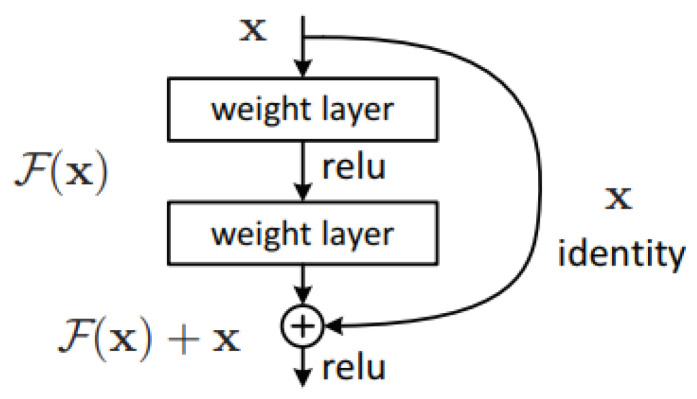
Building block of ResNet [19].

Residual networks (ResNets) [19] consist of reformulated convolutional layers that are learning residual functions with reference to the inputs. The authors argue that this type of networks are easier to optimize and can be of significantly increased depth. The implementation of a “residual block”, as described in [19], is straightforward: for every few convolutional layers a “shortcut connection” is added that runs parallel to these layers and implements the identity mapping. The output of the convolutional layers is then added to the output of the shortcut branch and the result is propagated to the subsequent block (Figure 4). Beside the use of shortcut connections, the network architecture is mainly inspired by the philosophy of VGG networks. All convolutional layers have small kernels of size 3×3 and follow two simple design rules: (i) for the same output feature map size, the layers have the same number of filters; (ii) when the feature map size is halved (with convolutional layers of stride 2), the number of filters is doubled so as to preserve the time complexity per layer. The authors tested architectures of varying depth in the range between 34 and 152 layers.

## 3. Experimental Results

In this work we aim to explore the performance of deep convolutional neural networks, in the context of breast mass classification into benign or malignant, in the case of (a) training from scratch and (b) fine-tuning. For the performance evaluation two datasets were used:

DDSM-400

**Figure 5:**
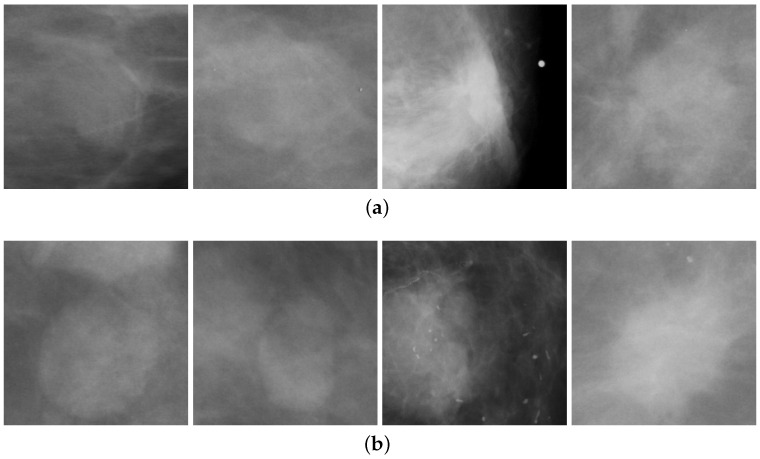
Two benign and two malignant samples from (**a**) the Curated Breast Imaging Subset of DDSM (CBIS-DDSM) and (**b**) DDSM-400.

This dataset consists of 400 mass ROIs extracted from the Digital Database for Screening Mammography (DDSM) [20] that was developed and used in our previous work [21]. The selected dataset was enriched due to a further processing of the ROIs, performed by expert radiologists, in order to acquire an accurate mass contour delineation using a semi-automatic segmentation method [22]. Two benign and two malignant samples from the dataset are depicted in Figure 5a.

CBIS-DDSM

The Curated Breast Imaging Subset of DDSM (CBIS-DDSM) [23] is an updated and standardized version of DDSM. It contains 10,239 mammographic images, linked to normal, benign, and malignant cases, selected and curated by a trained mammographer. The images are converted to the DICOM format and an updated ROI segmentation is provided for each lesion. The dataset is split to training and testing subsets to facilitate the direct comparison of performance between different methodologies. For this study, only cases concerning masses where extracted totaling 1319 and 378 ROIs for training and testing, respectively. Two benign and two malignant samples from the dataset are depicted in Figure 5b.

For the experiments concerning training networks from scratch the Glorot initialization [24] method was used. For the fine-tuning experiments the networks were initialized with the pre-trained weights from the ImageNet dataset [25]. The fully connected layers of the pre-trained networks were removed and replaced with randomly initialized ones. The final layer of each network is fully connected, containing two softmax neurons for the binary classification problem at hand. For the training process the Adam optimization method [26] was employed. Multiple learning rates were tested for each network at each scenario, which varied between $10^{-4}$ and $10^{-7}$. The batch size varied from 10 to 32 images, constrained by the memory requirements of each configuration. Early stopping was also used, terminating the training process if the AUC on the validation set did not improve for 15 consecutive epochs. The input image size was set to 224×224. Table 1 summarizes the training parameters used for each network.

The evaluation process for the DDSM-400 dataset is conducted employing 10-fold cross validation. Specifically, the dataset is partitioned randomly into 10 non-overlapping subsets of 40 samples. For each folding, the remaining 360 samples are further split to training (80%) and validation (20%) sets in a random fashion. The results provided for this dataset are calculated as the average of the ten runs. For the CBIS-DDSM dataset we used the original training partitioning which, similarly to DDSM-400, was randomly split to a new training set and a validation set.

The extraction of ROIs from the mammograms was performed by cropping a window of fixed size (1024×1024) for all lesions, centered around the mass. The image size was selected as such in order to be sufficient for all the masses in the dataset. In this way, resize-induced distortion is avoided while sufficient adjacent tissue is included for learning features in larger scales.

Data augmentation is an important part of the training process of deep networks. Through data augmentation, artificial training samples are generated by transforming existing images. The transformations used in our experimentation are rotation and flipping. In this way, meaningful samples are generated implying rotation invariance for the learned features. The data augmentation process is performed online, i.e., for each training sample random rotation and flipping is applied.

Table 2 and Table 3 summarize the performance of multiple networks, for the fine-tuning and from-scratch training scenarios, respectively. The metrics used for the performance evaluation are the area under ROC curve (AUC) and the classification accuracy (ACC).

**Figure 6:**
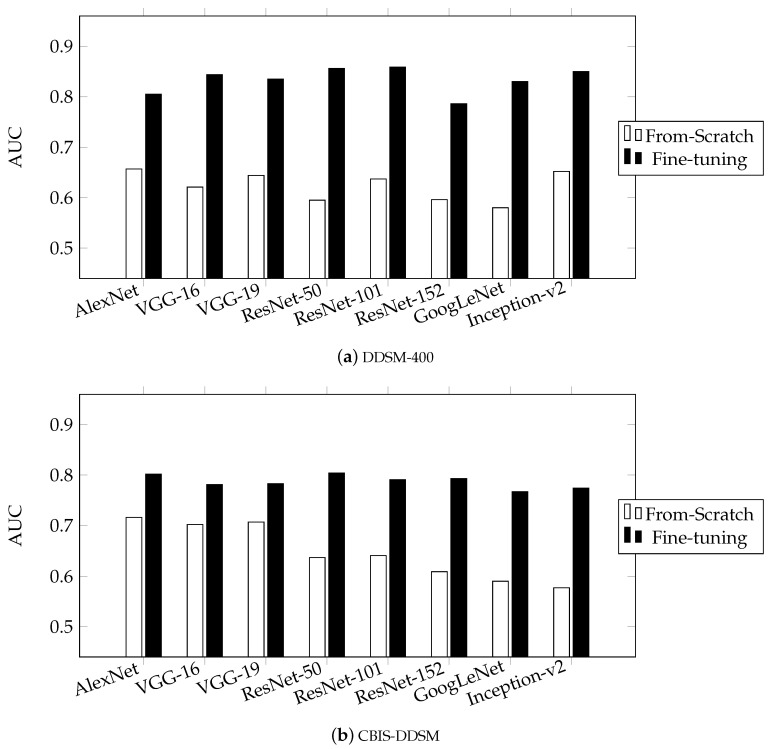
Performance of convolutional neural networks for from-scratch and fine-tuning scenarios in terms of AUC for (**a**) DDSM-400 and (**b**) CBIS-DDSM.

As shown in Table 2 and Table 3 and Figure 6, CNNs trained under the fine-tuning scenario achieved better performance compared with the ones trained from scratch. This confirms the tendency in state-of-the-art works to show a preference to the fine-tuning scenario. This difference in performance is attributed to the fact that the examined networks are designed and optimized for datasets several orders of magnitude larger than the available mammographic ones. However, once the network is trained, the learned features can address a variety of problems different in nature, after a fine-tuning stage.

Maximum performance was achieved using fine-tuning in ResNet-50 and ResNet-101 in both datasets. This coincides with the results reported in ILSVCR competition [27] as well. A counter-intuitive outcome of our investigation is that ResNets achieve low performance in the from-scratch training scenario, compared to AlexNet and VGG, which are of much higher capacity (Table 1), which is known to lead in over-fitting for small datasets. A possible cause for this is the increased complexity of ResNet, as it is an order of magnitude deeper than the other networks studied in our paper. As convolutional neural networks become deeper, i.e., with larger number of layers, the number of non-linearities is also increased, which results in more difficult convergence and increases the possibility of over-fitting. In the case of from-scratch training, maximum performance was achieved with AlexNet, which is the simpler and shallower network tested. This confirms the hypothesis that, when it comes to datasets with limited numbers of samples, such as the ones available for medical applications and specifically mammography, effective training of larger and complex networks cannot be achieved.

Table 4 shows the performance reported in three state-of-the-art methods, which exploit hand-crafted features for breast cancer diagnosis. The top-performing networks achieve marginally increased performance from [21] and surpass all the other works, when evaluated on the same dataset, in terms of AUC. However, the architecture developed in [21] utilizes a detailed lesion segmentation, provided as input, to extract shape features. Consequently, the performance of the system is highly dependent on the quality of the provided segmentation, that can be a task that requires significant time and effort spent from the user. In contrast, the lack of any dependencies in CNN results in a more concrete end-to-end classification system where diagnosis can be fully automated.

## 4. Discussion and Concluding Remarks

In this paper, the use of deep convolutional neural networks for breast cancer diagnosis from mammograms with mass lesions is investigated. The performance of various networks is assessed on two digitized mammogram databases of different sizes. The first database comprises 400 images from DDSM while the other comprises 1696. Two scenarios of training are considered: from-scratch, where the weights of the network are initialized from random distributions, and fine-tuning, where the training is initialized by weights of a network that has already been trained using another dataset.

From-scratch training is a tedious process as it requires the convergence of all the network parameters, starting from a random state. On the one hand, increasing the depth of networks leads to better features in terms of discriminating ability; on the other hand, increasing depth makes the network prone to various adverse effects, such as vanishing or exploding gradients and overfitting. Although several improvements of the training process and network architecture have been proposed to eliminate the complications imposed by the growing capacity and complexity of the models, a major requirement for an effective training is still a large amount of data, which is not available for most medically oriented applications, such as the problem at hand, i.e., breast cancer diagnosis in mammograms.

In contrast, a fine-tuning training scenario for a CNN is naturally a simpler process, and puts emphasis on domain-specific features rather than generic low-level ones. In this way it applies major corrections of network parameters only for layers that are closer to the end of the network, where higher-level concepts are captured.

Having that in mind, we would like to emphasize the need for assembling and constructing large-scale mammographic datasets to support the active research fields of computer-aided detection and diagnosis, specifically aimed for more advanced imaging modalities such as digital mammography, which is the current standard and tomosynthesis that has an emerging role for imaging specific patient groups [30].

## Figures and Tables

**Table 1:** Network parameters in the case of fine-tuning (FT) and from-scratch (SC) training scenarios.

CNN	Number of Weights	Batch Size	Learning Rate			Best Model Iter.	
			FT	SC		FT	SC
AlexNet	56,866,848	32	$10^{-5}$	$10^{-5}$		6	65
VGG-16	134,256,320	32	$10^{-5}$	$10^{-5}$		9	58
VGG-19	139,564,736	32	$10^{-5}$	$10^{-5}$		14	64
ResNet-50	23,512,128	32	$10^{-5}$	$10^{-4}$		4	104
ResNet-101	42,504,256	16	$10^{-5}$	$10^{-5}$		13	92
ResNet-152	58,147,904	10	$10^{-5}$	$10^{-7}$		31	38
GoogLeNet	10,299,840	32	$10^{-5}$	$10^{-5}$		12	12
Inception-BN (v2)	16,072,832	32	$10^{-4}$	$10^{-5}$		43	108

**Table 2:** Performance of deep neural networks using the from-scratch training scenario.

CNN	DDSM-400			CBIS-DDSM	
	AUC	ACC		AUC	ACC
AlexNet	0.657	0.610		0.716	0.656
VGG-16	0.621	0.590		0.702	0.580
VGG-19	0.644	0.588		0.707	0.581
ResNet-50	0.595	0.548		0.637	0.627
ResNet-101	0.637	0.588		0.641	0.662
ResNet-152	0.596	0.543		0.609	0.647
GoogLeNet	0.580	0.569		0.590	0.598
Inception-BN (v2)	0.652	0.590		0.577	0.654

**Table 3:** Performance of convolutional neural networks (CNNs) initialized on pre-trained weights (fine-tuning).

CNN	DDSM-400			CBIS-DDSM	
	AUC	ACC		AUC	ACC
AlexNet	0.805	0.733		0.802	0.753
VGG-16	0.844	0.748		0.781	0.716
VGG-19	0.835	0.738		0.783	0.736
ResNet-50	0.856	0.743		0.804	0.749
ResNet-101	0.859	0.785		0.791	0.753
ResNet-152	0.786	0.630		0.793	0.755
GoogLeNet	0.830	0.758		0.767	0.720
Inception-BN (v2)	0.850	0.780		0.774	0.747

**Table 4:** Performance of state-of-the-art methods based on hand-crafted features.

Method	DDSM-400	
	AUC	ACC
Tsochatzidis et al. [21]	0.85	0.81
Rouhi et al. [28]	0.78	0.79
Xie et al. [29]	0.72	0.68
